# Prognostic value of circulating amino-terminal pro-C-type natriuretic peptide in critically ill patients

**DOI:** 10.1186/cc10007

**Published:** 2011-01-31

**Authors:** Alexander Koch, Sebastian Voigt, Edouard Sanson, Hanna Dückers, Andreas Horn, Henning W Zimmermann, Christian Trautwein, Frank Tacke

**Affiliations:** 1 Department of Medicine III, RWTH-University Hospital Aachen, Pauwelsstrasse 30, 52074 Aachen, Germany

## Abstract

**Introduction:**

C-type natriuretic peptide (CNP) is a paracrine molecule which is mainly synthesized in the vasculature. High levels have been reported in sepsis, and CNP has been proposed as a biomarker predicting sepsis in traumatized patients. We aimed at evaluating the diagnostic and prognostic value of N-terminal pro-CNP (NT-proCNP) for predicting sepsis, disease severity and mortality in critically ill medical patients.

**Methods:**

273 critically ill patients (197 patients with sepsis or septic shock, 76 without evidence of sepsis) and 43 healthy controls were consecutively included in a prospective clinical single-center non-interventional study at the Medical Intensive Care Unit, RWTH-University Aachen, Germany. Patients' outcome was followed for about 1 year. NT-proCNP serum concentrations were determined upon ICU admission, as well as in the mornings of day 3 and day 7 after admission. Intensive care treatment measures as well as routine and experimental laboratory parameters were recorded and analyzed.

**Results:**

NT-proCNP serum concentrations upon admission to the ICU were elevated in critically ill patients as compared with healthy controls. Patients with sepsis had significantly higher NT-proCNP levels than non-sepsis patients. NT-proCNP was strongly associated with inflammatory parameters (i.e. C-reactive protein, procalcitonin and TNF-α), biomarkers of organ dysfunction and clinical composite scores (APACHE-II, SOFA, SAPS2). NT-proCNP levels at admission and day 3 were found to be a strong predictive marker for ICU- and overall survival. Moreover, a decline of serum NT-proCNP after admission to the ICU was associated with reduced mortality. The predictive power of serum NT-proCNP was similar to 'conventional' prognostic tools such as clinical scores.

**Conclusions:**

NT-proCNP is significantly elevated in critically ill patients, with highest levels in sepsis. Inflammation as well as organ function are strongly associated with NT-proCNP serum concentrations. Low initial NT-proCNP levels and a decline during initial treatment indicate a favourable ICU- and long-term outcome.

## Introduction

The natriuretic peptide family consists of three distinctive members: atrial natriuretic peptide (ANP), brain natriuretic peptide (BNP) and C-type natriuretic peptide (CNP). These peptides exert multiple potent diuretic, natriuretic and vasorelaxant functions, thereby directly influencing body-fluid homeostasis and blood pressure control [1,2]. As ANP and BNP are mainly derived from the heart in response to atrial and ventricular stretching, they have been thought to act as cardiac hormones and linked to cardiac dysfunction [2,3]. In patients with severe sepsis, BNP has been proposed as a useful biomarker to predict survival [4,5], most likely by indicating septic myocardial depression [4,5].

CNP is synthesized as a precursor proCNP protein, and conversion of proCNP to the biologically active hormone CNP is processed by the intracellular endoprotease furin [6]. Amino-terminal pro-C-type natriuretic peptide (NT-proCNP) is the N-terminal fragment of the C-type natriuretic peptide precursor. As a cleavage product of proCNP, NT-proCNP circulates in equimolar amounts with CNP in human plasma and is considered to be a more reliable marker of the extent of CNP biosynthesis [7]. Due to its extra-cardiac origin and its high expression in the brain, CNP was initially believed to be a neuropeptide, involved in central regulatory mechanisms [8,9]. At present it is known that CNP is widely expressed in various tissues, with particularly high concentrations in the vascular endothelium [10] and chondrocytes [11], inducing vasorelaxation and vascular remodeling, as well as regulating bone growth [12]. Compared with ANP and BNP, CNP exerts limited diuretic and natriuretic functions, but counteracts angiotensin II- or endothelin-1-induced vasoconstriction and complements the actions of other endothelial vasorelaxant mediators such as nitric oxide (NO) and prostacyclin [13]. IL-1, endotoxins and particularly TNF-α, which are increased in states of sepsis, can stimulate CNP release from isolated endothelial cells and in this way regulate local vascular tone [14]. CNP release in response to proinflammatory cytokines suggests an interaction of macrophageal cytokine synthesis and vascular endothelium [15]. This link indicates a potential pathophysiological role of CNP in sepsis and septic shock, which are characterized by arteriolar vasodilatation, hypotension, and inadequate tissue perfusion [16]. In a small cohort of patients with sepsis and septic shock, high serum CNP concentrations have been demonstrated [17]. Moreover, in a recent study, NT-proCNP has been proposed as a novel biomarker for predicting the development of sepsis in multiple trauma patients [18]. The diagnostic and prognostic value of NT-proCNP measurements in critically ill medical patients is currently unknown.

We therefore conducted a large study with critically ill patients in a medical ICU, performing longitudinal measurements of NT-proCNP serum concentrations during the first week of ICU treatment, to address whether NT-proCNP is activated in critical illness, whether NT-proCNP has diagnostic value for sepsis and/or multiorgan failure, and whether NT-proCNP can serve as a prognostic predictor for ICU and long-term survival.

## Materials and methods

### Study design and patient characteristics

The study protocol was conducted in accordance with the ethical standards laid down in the Declaration of Helsinki and approved by the local ethics committee (ethics committee of the University Hospital Aachen, RWTH-University, Aachen, Germany, reference number EK 150/06). We investigated 273 patients (172 male, 101 female with a median age of 64 years; range 18 to 90 years) who were admitted consecutively to the General Internal Medicine ICU at the RWTH-University Hospital Aachen, Germany (Table 1). Written informed consent was obtained from the patient, his or her spouse, or the appointed legal guardian. Patients that were expected to have a short-term (< 72 hours) intensive care treatment due to post-interventional observation or acute intoxication were not included in this study [19]. Medium length of stay at the ICU was nine days (range 1 to 137 days) and medium length of stay in hospital was 27 days (range 2 to 151 days).

**Table 1:** Disease etiology of the study population

	Sepsis	Non-sepsis
	*n *= 197	*n *= 76
**Etiology of sepsis critical illness**		
Site of infection n (%)		
pulmonary	117 (60%)	
abdominal	30 (15%)	
urogenital	10 (5%)	
others	40 (20%)	
**Etiology of non-sepsis critical illness**		
n (%)		
decompensated liver cirrhosis		19 (25%)
cardio-pulmonary disease		25 (33%)
others		32 (42%)

We prospectively collected patient data, clinical information and blood samples. The clinical course of patients was observed in a follow-up period by directly contacting the patients, the patients' relatives, or their primary care physicians. Critical care patients were divided upon ICU admission into two categories: sepsis patients and non-sepsis patients. Patients in the sepsis group met the criteria proposed by the American College of Chest Physicians and the Society of Critical Care Medicine Consensus Conference Committee for severe sepsis and septic shock [20].

The control group consisted of 43 healthy blood donors (28 male, 15 female; median age 53 years, range 24 to 68 years) from the local blood transfusion institute at the University Hospital Aachen. At our blood transfusion institute, all volunteers that donate blood agreed (after informed consent) to contribute to ongoing biomarker studies. All control subjects had normal values for blood counts, C-reactive protein, and liver enzymes, and tested negative for hepatitis B and C and HIV.

In addition, ICU patients were divided according to their Acute Physiology and Chronic Health Evaluation (APACHE) II score into patients with "moderate disease severity" (APACHE-II < 10, with a statistical risk of death below 10%) and "high disease severity" (APACHE-II > 10) [21].

### Characteristics of sepsis and non-sepsis patients

Among the 273 critically ill patients enrolled in this study, 197 patients conformed to the criteria of bacterial sepsis (Table 1). Non-sepsis patients were admitted to the ICU mainly due to cardiopulmonary diseases (myocardial infarction, pulmonary embolism, and cardiac pulmonary edema), decompensated liver cirrhosis, or other critical conditions, and did not differ in age or sex from sepsis patients (Table 1). Compared with the cohort of non-sepsis patients, sepsis patients were more often in need of mechanical ventilation in the longer term (Table 2) and displayed significantly higher levels of routinely used biomarkers of inflammation (i.e. C-reactive protein, procalcitonin, white blood cell count; data not shown). Both groups did not differ in APACHE-II score and simplified acute physiology score (SAPS) 2, vasopressor demand, or laboratory parameters indicating liver or renal dysfunction (data not shown).

**Table 2:** Baseline patient characteristics and NT-proCNP serum concentrations

Parameter	All patients	Sepsis	Non-sepsis
Number	273	197	76
Sex (male/female)	172/101	128/69	44/32
Age median (range) (years)	64 (18-90)	65 (20-90)	60 (18-85)
APACHE-II score median (range)	17 (2-40)	18 (3-40)	15 (2-31)
SAPS2 score median (range)	44 (0-80)	44.5 (0-79)	41.5 (13-80)
ICU days median (range)	9 (0-137)	12 ** (0-137)	6 ** (1-45)
Hospital days median (range)	27 (2-151)	30 ** (2-151)	14 ** (2-85)
Death during ICU n (%)	76 (27.8%)	61 (31.0%)	15 (19.7%)
Death during follow up n (%)	132 (50.2%)	101 (53.2%)	31 (42.5%)
Mechanical ventilation n (%)	194 (73.2%)	144 (75%)	50 (68.5%)
Ventilation time median (range) (hours)	126 (0-2966)	180 * (0-2966)	48.5 (0-986)
Pre-existing diabetes n (%)	88 (33.1%)	60 (31.3%)	28 (37.8%)
BMI median (range) (m²/kg)	25.8 (14.0-66.7)	25.9 (14.0-66.7)	25.8 (15.9-53.3)
NT-proCNP day 1 median (range) (pmol/L)	4.07 (0-42)	5.6 (0-42) **	1.48 (0-42) **
NT-proCNP day 3 median (range) (pmol/L)	4.79 (0-42)	5.81 (0-42) *	0.90 (0-42) *
NT-proCNP day 7 median (range) (pmol/L)	3.91 (0-42)	4.59 (0-42)	2.37 (0-41.34)

APACHE, Acute Physiology and Chronic Health Evaluation; BMI, body mass index; NT-proCNP, amino-terminal pro-C-type natriuretic peptide; SAPS, simplified acute physiology score.

Long-term follow-up data on survival were only available in 263 of 273 patients.

Significant differences between sepsis and non-sepsis patients are marked by *(*P *< 0.05) or **(*P *< 0.001).

### NT-proCNP measurements

Prior to therapeutic interventions, blood samples were collected upon admission to the ICU, as well as in the morning of day three and seven after admission. Following centrifugation at 2000 g at 4°C for 10 minutes, serum and plasma aliquots of 1 mL were frozen immediately at -80°C. NT-proCNP serum concentrations were analyzed using a specific commercial enzyme immunoassay (#BI-20872, BioMedica, Vienna, Austria; distributor: Immundiagnostik AG, Bensheim, Germany). Intra-assay coefficient of variation was 5.3 to 8.3%, and inter-assay coefficient of variation was 7 to 9%. IL-6, IL-10, TNF-α (all Siemens Healthcare, Erlangen, Germany), and procalcitonin (Kryptor, B.R.A.H.M.S. Diagnostica, Henningsdorf, Germany) were measured by commercial chemiluminescence assays, following manufacturers' instructions.

### Statistical analysis

Data are given as median and range due to the skewed distribution of most of the parameters. Differences between two groups were assessed by Mann-Whitney-*U*-test and multiple comparisons between more than two groups have been conducted by Kruskal-Wallis analysis of variance and Mann-Whitney-*U*-test for *post hoc *analysis. Box plot graphics illustrate comparisons between subgroups and they display a statistical summary of the median, quartiles, range, and extreme values. The whiskers extend from the minimum to the maximum value excluding outside and far out values, which are displayed as separate points. An outside value (indicated by an open circle) was defined as a value that is smaller than the lower quartile minus 1.5-times interquartile range, or larger than the upper quartile plus 1.5-times the interquartile range. A far out value (indicated by an asterisk) was defined as a value that is smaller than the lower quartile minus three times interquartile range, or larger than the upper quartile plus three times the interquartile range [22]. All values, including "outliers", have been included for statistical analyzes. The Wilcoxon signed-rank test was applied as a non-parametric statistical hypothesis test for comparing repeated measurements (days one, three and seven) in the same individuals. Correlations between variables have been analysed using the Spearman correlation tests, where values of *P *< 0.05 were considered statistically significant [23]. The prognostic value of the variables was tested by univariate and multivariate analyses in the Cox regression model. Kaplan-Meier curves were plotted to display the impact on survival [24]. After significant results from the univariate and multivariate Cox regression analyses, Kaplan-Meier curves and log-rank test calculations were performed subsequently for different cut-off values for NT-proCNP (5, 6, 7, 8, 9, 10, 11, and 12 pmol/L). The threshold of 8 pmol/L yielded highest log-rank values. Receiver operating characteristic (ROC) curve analysis and the derived area under the curve (AUC) statistic provides a global and standardized appreciation of the accuracy of a marker or a composite score for predicting an event. ROC curves were generated by plotting sensitivity against 1-specificity. All statistical analyses were performed with SPSS version 12.0 (SPSS, Chicago, IL, USA).

## Results

### NT-proCNP serum concentrations upon admission to the ICU are elevated in critically ill patients as compared with healthy controls

**Figure 1:**
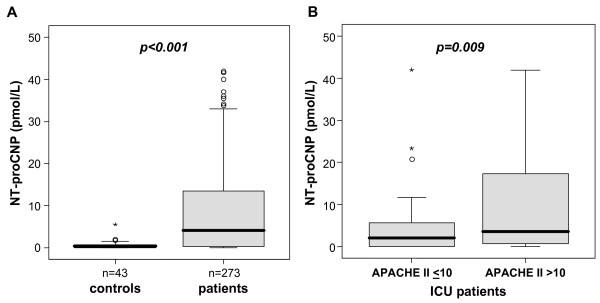
**Serum NT-proCNP concentrations in critically ill patients at ICU admission**. **(a) **Serum amino-terminal pro-C-type natriuretic peptide (NT-proCNP) concentrations at admission to the medical ICU are significantly (*P *< 0.001, U-test) elevated in critically ill patients (*n *= 273) as compared with healthy controls (*n *= 43). **(b) **Serum NT-proCNP concentrations at admission to the medical ICU are significantly (*P *= 0.009, U-test) elevated in critically ill patients with high initial Acute Physiology and Chronic Health Evaluation (APACHE) II scores (> 10) in comparison to patients with low APACHE-II scores (</=10). Box plot are displayed, where the bold line indicates the median per group, the box represents 50% of the values, and horizontal lines show minimum and maximum values of the calculated non-outlier values; asterisks and open circles indicate outlier values.

We first tested whether NT-proCNP is activated in critically ill patients. Upon admission to the medical ICU, critically ill patients displayed significantly higher NT-proCNP serum concentrations as compared with healthy controls (median 0.34 pmol/L, range 0 to 5.53, in controls versus 4.07 pmol/L, range 0 to 42, in ICU patients, *P *< 0.001; Figure 1a). Moreover, NT-proCNP levels were significantly higher in patients with APACHE-II scores above 10 in comparison to ICU patients admitted with APACHE-II scores of 10 or less (Figure 1b), indicating that NT-proCNP is further related to the disease severity.

### NT-proCNP serum concentrations indicate sepsis and organ failure in medical ICU patients

**Figure 2:**
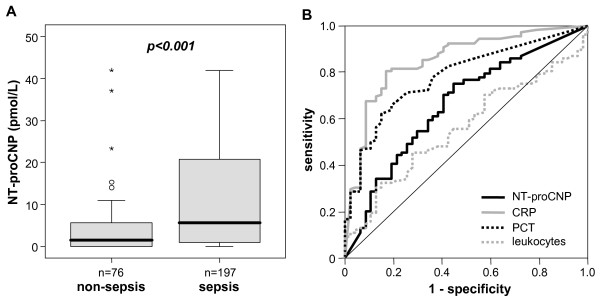
**Serum NT-proCNP concentrations in critically ill patients are elevated in sepsis**. **(a) **In patients with sepsis amino-terminal pro-C-type natriuretic peptide (NT-proCNP) serum concentrations are significantly (*P *< 0.001, U-test) higher as compared with patients with non-septic etiology of critical illness. Box plot are displayed, where the bold line indicates the median per group, the box represents 50% of the values, and horizontal lines show minimum and maximum values of the calculated non-outlier values; asterisks and open circles indicate outlier values. **(b) **Receiver operating characteristic (ROC) curve analyses comparing the diagnostic power in predicting sepsis of NT-proCNP in critically ill patients in a medical ICU (black line, area under the curve (AUC) = 0.661) with classical markers of inflammation and bacterial infection: C-reactive protein (CRP; grey line, AUC = 0.852), procalcitonin (PCT; dotted black line, AUC = 0.783), and white blood cell count (leucocytes; dotted grey line, AUC = 0.560).

Based on a recent study that suggested NT-proCNP as a novel biomarker for predicting sepsis in trauma patients [18], we tested whether serum NT-proCNP might identify patients with sepsis in the medical ICU setting as well. We found significantly elevated NT-proCNP serum concentrations in septic patients versus patients with non-septic etiology of critical illness (median 1.48 pmol/L in non-sepsis patients versus 5.60 pmol/L in sepsis patients; Figure 2a and Table 2). We next compared the diagnostic accuracy of NT-proCNP with classical, routinely used markers of inflammation and bacterial infection by using ROC curve analyses. C-reactive protein (CRP) and procalcitonin (PCT) achieved AUC statistics of 0.852 and 0.783, respectively, while NT-proCNP and white blood cell count only reached AUC values of 0.661 and 0.560, respectively (Figure 2b). Although our data demonstrated a strong elevation of NT-proCNP in critically ill patients upon admission to the ICU, NT-proCNP itself evidenced inferior diagnostic accuracy for sepsis as compared with classical biomarkers.

**Figure 3:**
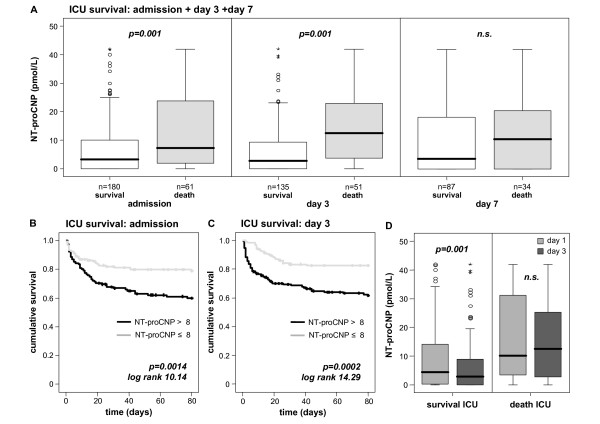
**Association of sequentially measured NT-proCNP concentrations with ICU mortality**. **(a) **Patients that die during the course of ICU treatment have significantly higher serum amino-terminal pro-C-type natriuretic peptide (NT-proCNP) levels on admittance to the ICU (*P *= 0.001) and on day 3 (*P *= 0.001) than survivors. At day 7 no significant changes in NT-proCNP levels between survivors and non-survivors were detected. Box plot are displayed, where the bold line indicates the median per group, the box represents 50% of the values, and horizontal lines show minimum and maximum values of the calculated non-outlier values; asterisks and open circles indicate outlier values. **(b and c) **Kaplan-Meier survival curves of ICU patients are displayed, showing that patients with high NT-proCNP levels (> 8 pmol/L, grey) have an increased short-term mortality in the ICU as compared with patients with low NT-proCNP serum concentrations. *P*-values are given in the figure. **(d) **Survivors in the ICU display a significant decrease in NT-proCNP serum concentrations between day 1 and day 3 (*P *= 0.001) as compared with non-survivors.

At admission to the ICU, serum NT-proCNP concentrations in the total cohort and the subgroup of sepsis patients were closely correlated to markers of inflammation and bacterial infection, such as PCT, CRP and TNF-α (Table 3). We could also reveal strong associations with renal and hepatic functions in the total cohort of critically ill patients and in sepsis patients. In particular, we could show a close association with renal function as displayed by highly significant correlations with creatinine, urea, and cystatin C serum concentrations and the glomerular filtration rate of cystatin C (Table 3), representing potential renal clearance of NT-proCNP in critically ill patients. Serum NT-proCNP concentrations were inversely correlated to parameters reflecting hepatic biosynthetic capacity, namely albumin (Figure 3c) and pseudocholinesterase activity (Table 3).

**Table 3:** Correlations with NT-proCNP serum concentrations at admission.

	All patients		Sepsis		Non-sepsis	
			
Parameters	r	*P*	r	*P*	r	*P*
						
***Markers of inflammation***						
Leukocytes	0.178	0.006	0.196	0.011	-	n.s.
CRP	0.296	< 0.001	0.205	0.008	-	n.s.
Procalcitonin	0.456	< 0.001	0.398	< 0.001	0.316	0.031
IL-6	0.188	0.047	-	n.s.	-	n.s.
TNF-α	0.500	< 0.001	0.444	< 0.001	0.578	0.001
						
***Markers of organ function***						
Creatinine	0.715	< 0.001	0.746	< 0.001	0.605	< 0.001
Urea	0.648	< 0.001	0.684	< 0.001	0.459	< 0.001
Cystatin C	0.700	< 0.001	0.757	< 0.001	0.419	0.011
Cystatin C GFR	-0.707	< 0.001	-0.749	< 0.001	-0.749	0.002
AP	0.441	0.012	-	n.s.	-	n.s.
PCHE	-0.279	< 0.001	-0.214	0.009	-0.310	0.015
Albumin	-0.329	< 0.001	-0.311	0.001	-	n.s.
						
***Clinical scores***						
APACHE II	0.206	0.006	0.388	0.001	-	n.s.
SOFA	0.261	0.026	-	n.s.	-	n.s.
SAPS2	0.230	0.015	0.361	0.001	-	n.s.

r, correlation coefficient; *P*, *P*-value; r and *P*-values by Spearman rank correlation. AP, alkaline phosphatase; APACHE, Acute Physiology and Chronic Health Evaluation; CRP, C-reactive protein; GFR, glomerular filtration rate; IL-6, interleukin 6; NT-proCNP, amino-terminal pro-C-type natriuretic peptide; PCHE, pseudocholinesterase; SAPS2, simplified acute physiology score; SOFA, sequential organ failure assessment; TNF-α, tumor necrosis factor α.

For the total cohort of critically ill patients, as well as for sepsis patients, we found a strong association of NT-proCNP serum concentrations at admission to the ICU and established clinical scores like APACHE II, sequential organ failure assessment (SOFA) and SAPS2 (Table 3). These findings suggest that NT-proCNP levels are linked to disease severity in critical illness and in sepsis.

### NT-proCNP is a strong predictive marker for ICU and overall survival in critically ill patients, and a decline of NT-proCNP levels after admission to the ICU is associated with a favorable outcome

Based on the clear associations between NT-proCNP, inflammatory markers, organ dysfunction and prognostic clinical scores, we hypothesized that NT-proCNP measurements could predict mortality in critically ill medical patients. We determined NT-proCNP serum concentrations at ICU admission, and at days three and seven of ICU treatment. Its prognostic impact on ICU and overall survival among all critically ill patients and the subgroups of sepsis and non-sepsis patients was assessed over a long-term follow-up period (median observation time 348 days, range 29 to 884 days).

Patients that died during the subsequent ICU treatment showed significantly higher NT-proCNP levels at admission and on day three (Figure 3a and Table 4). On day seven, a trend to higher NT-proCNP levels in patients who died in the ICU could be observed, but did not reach statistical significance. In order to account for the potential impact of volume load during ICU treatment on NT-proCNP levels, we also normalized NT-proCNP concentrations to the patients' current hematocrit levels, revealing the same findings as for NT-proCNP by itself (Table 5).

**Table 4:** NT-proCNP serum concentrations and association with survival

	NT-proCNP median (range) (pmol/L)		
	
Outcome	Admission	Day 3	Day 7
*Survivor ICU*	3.28 (0-42)	2.75 (2.3-20)	3.55 (3.6-20)
*Death ICU*	7.32 (3.4-20)	12.49 (3.2-20)	10.51 (5.6-20)
*Survivor overall*	2.53 (2.31-20)	2.62 (2.33-20)	3.32 (3.67-20)
*Death overall*	6.64 (0-20)	7.86 (3.10-20)	8.94 (5.38-20)

NT-proCNP, amino-terminal pro-C-type natriuretic peptide.

**Table 5:** NT-proCNP serum concentrations (normalized to hematocrit) and association with survival

	NT-proCNP/hematocritmedian (range)		
	
Outcome	Admission	Day 3	Day 7
*Survivor ICU*	9.92 (0-191)	9.30 (0-175)	11.30 (0-191)
*Death ICU*	21.35 (0-221)	39.02 (0-200)	36.24 (0-168)
*Survivor overall*	7.67 (0-191)	9.04 (0-175)	9.51 (0-175)
*Death overall*	20.0 (0-221)	22.60 (0-200)	29.79 (0-168)

NT-proCNP, amino-terminal pro-C-type natriuretic peptide.

Moreover, low NT-proCNP levels upon admission to the ICU and on day three were a strong prognostic predictor for ICU survival (admission *P *= 0.001, day three *P *= 0.001, day seven not significant; Cox regression analyses). In this respect, NT-proCNP levels showed comparable prognostic accuracy like established multifactorial scores such as SOFA or the SOFA score change during the first three days of ICU treatment (AUC = 0.711 for NT-proCNP, 0.684 for SOFA, and 0.646 for SOFA score changes in ROC analyses). Kaplan-Meier curves showed, using a cut-off value for serum NT-proCNP of 8 pmol/L, significantly improved ICU survival for critically ill patients with low NT-proCNP levels at admission and on day three (Figures 3b and 3c, and Table 4). Interestingly, survivors displayed a significant decrease in NT-proCNP serum concentrations from admission to day three (*P *= 0.001; Figure 3d), while NT-proCNP levels remained stably elevated in non-survivors.

In multivariate Cox regression analyses for variables obtained at ICU admission including CRP and PCT as markers of inflammation and infection, NT-proCNP remained an independent significant prognostic parameter. However, if markers of hepatic and renal dysfunction (albumin and creatinine) were included, NT-proCNP did not reach independent prognostic significance (detailed data not shown).

**Figure 4:**
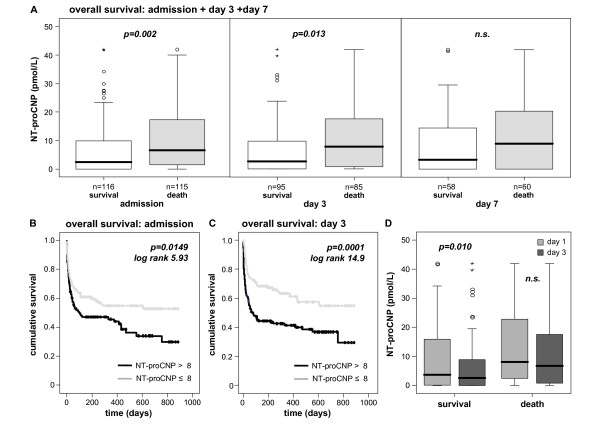
**Association of sequentially measured NT-proCNP concentrations with long-term mortality**. **(a) **Serum amino-terminal pro-C-type natriuretic peptide (NT-proCNP) concentrations are significantly associated with the overall survival of critically ill patients. Survivors have significantly lower serum NT-proCNP levels on admittance to the ICU (*P *= 0.002) and on day 3 (*P *= 0.013). Box plot are displayed, where the bold line indicates the median per group, the box represents 50% of the values, and horizontal lines show minimum and maximum values of the calculated non-outlier values; asterisks and open circles indicate outlier values. **(b and c) **Kaplan-Meier survival curves of ICU patients are displayed, showing increased overall mortality in the long-term follow up of patients with high NT-proCNP levels (on admission > 8 pmol/L, grey) as compared with patients with low NT-proCNP serum concentrations. *P*-values are given in the figure. **(d) **Likewise in patients surviving ICU treatment, long-term survivors showed a significant decline of NT-proCNP serum between day 1 and day 3 (*P *= 0.010).

Although the long-term outcome of critically ill patients is certainly affected by manifold factors, we also tested whether NT-proCNP levels during the early course of ICU treatment could predict the long-term survival. Patients that will die during long-term follow up had significantly higher NT-proCNP levels than survivors at ICU admission and day three (Figure 4a). By Cox regression analyses, high NT-proCNP levels at admission (*P *= 0.002) and day three (*P *= 0.013) predicted long-term mortality in critically ill patients. We also observed a trend to high levels predicting mortality for NT-proCNP measured at day seven. Using Kaplan-Meier curves, with cut-off values for serum NT-proCNP of 8 pmol/L, we demonstrated significantly improved overall survival for critically ill patients with low NT-proCNP (Figures 4b and 4c; Table 4).

In an analogous manner as for short-term survival, a decrease in NT-proCNP serum concentrations from day one to three was associated with a favorable long-term prognosis. This was displayed by a significant decline in NT-proCNP levels from admission to day three in survivors (*P *= 0.010, Figure 4d), but not in non-survivors.

## Discussion

NT-proCNP has been recently proposed as a novel diagnostic marker for sepsis in traumatized patients without traumatic brain injury [18]. It was suggested that NT-proCNP levels above a range of 1.6 to 3.1 pmol/L identified sepsis with high sensitivity and specificity in these patients [18]. However, the limited number of investigated patients, as well as the missing evaluation of renal function and confounding factors such as age, gender, and body mass index, have been criticized and regarded as limitations of this prior study [25].

In our study, we confirmed that NT-proCNP is activated in critically ill medical patients. In accordance with previous data [17,18], sepsis patients showed significantly higher NT-proCNP levels than non-sepsis patients. However, when we compared the diagnostic accuracy of NT-proCNP levels with routinely used biomarkers of inflammation and infection, such as CRP, PCT, and white blood cell count [26,27], NT-proCNP displayed inferior diagnostic precision for sepsis as compared with classical parameters in medical ICU patients.

In animal models, the regulation of CNP depends on a variety of influencing factors such as glucocorticoids, thyroid hormones, vasoactive peptides, and catabolic state [28-32], but very little is known about regulatory mechanisms in states of critical illness in humans. Using correlation analyses, our study revealed significant associations between NT-proCNP and established laboratory biomarkers reflecting inflammation and organ function in medical ICU patients.

CNP itself is widely expressed in the vasculature, and highest concentrations are found in the endothelium [15]. Inflammatory cytokines such as IL-1, TNF-α, and endotoxin are known to trigger the release of CNP from endothelial cells in animal models [14], which fits well to the close correlation of serum NT-proCNP with serum TNF-α, PCT, CRP, and leukocytes in our cohort of critically ill patients. Of note, this association could not be observed for IL-6, although IL-6 serum levels were strongly increased in sepsis patients. However, we did not measure additional parameters of the NO and prostaglandin system such as nitrate, nitrite, prostacyclin, or thromboxane levels, because most of these parameters are rather unstable, even at 4°C, making an appropriate pre-analytic sample acquisition in the ICU setting with a large patient cohort difficult. We therefore cannot estimate the impact of these systems on the NT-proCNP levels in critical illness.

The pathophysiological consequences of high circulating NT-proCNP in critically ill patients are not clear at present. Very recently, CNP has also been described as a regulator of glucose metabolism by acting as an inhibitor of insulin action [33]. In a novel mouse model of inducible CNP-depletion, CNP-deficient mice were found to have reduced food intake, lower endogenous insulin levels, and a significantly reduced insulin tolerance [33]. Interestingly, NT-proCNP in critically ill patients was indeed correlated with endogenous insulin levels (C-peptide) as well as serum resistin and retinol-binding protein 4, two mediators of insulin resistance (data not shown) [23,34]. Further studies are needed to elucidate the potential pathogenic function of NT-proCNP, especially its possible involvement in promoting insulin resistance and metabolic dysregulation in the critically ill.

Our study is the first to demonstrate the prognostic value of NT-proCNP measurements in medical ICU patients. NT-proCNP serum concentrations upon admission as well as on day three were closely associated with ICU survival as well as long-term survival, and high NT-proCNP levels indicated an unfavorable prognosis. Surviving patients displayed an individual decline of NT-proCNP between days one and three. However, we would like to emphasize that the prognostic power of NT-proCNP as a biomarker is dependent on renal function as well as hepatic function, thereby limiting the general use of any static cut-off (e.g., 8 pmol/L as shown in Kaplan-Meier curves). Larger studies should evaluate whether adjustment of NT-proCNP to individual renal and hepatic functions might further improve its diagnostic power.

## Conclusions

NT-proCNP was significantly elevated in critically ill patients with highest levels in sepsis. Inflammation as well as organ function strongly impact NT-proCNP concentrations. Low NT-proCNP levels and a decline during initial treatment were associated with favorable ICU and long-term outcomes. The predictive power of serum NT-proCNP was similar to 'conventional' prognostic tools such as clinical scores. Yet, further studies are needed to elucidate the underlying pathomechanisms of NT-proCNP in patients with critical illness.

## Key messages

• NT-proCNP, a paracrine molecule released mainly from vasculature, has been implicated in inflammatory and metabolic pathways and has been recently proposed as a novel biomarker for predicting sepsis in traumatized patients.

• In critically ill medical patients, NT-proCNP serum concentrations upon ICU admission are elevated as compared with healthy controls and are higher in sepsis patients than in non-sepsis patients.

• In the initial course of ICU treatment, NT-proCNP serum concentrations significantly decline between admission and day three.

• NT-proCNP levels at admission to the ICU are correlated to biomarkers of inflammation, organ dysfunction, and disease severity.

• NT-proCNP is a strong outcome predictor, and a decline of NT-proCNP serum concentrations after ICU admission is associated with a reduced mortality.

## Abbreviations

ANP: atrial natriuretic peptide; APACHE: Acute Physiology and Chronic Health Evaluation; AUC: area under the curve; BNP: brain natriuretic peptide; CNP: C-type natriuretic peptide; CRP: C-reactive protein; IL: interleukin; NO: nitric oxide; NT-proCNP: amino-terminal pro-C-type natriuretic peptide; PCT: procalcitonin; ROC: receiver operating characteristic; SAPS: simplified acute physiology score; SOFA: sequential organ failure assessment; TNF-α: tumor necrosis factor α.

## Competing interests

The authors declare that they have no competing interests.

## Authors' contributions

AK, FT, and CT designed the study, analyzed data and wrote the manuscript. SV performed measurements. ES, HZ, HD, and AH collected data and assisted in patient recruitment.
